# Rescue of tomato yellow leaf curl virus mutants harboring heterologous iterons through *in planta* evolution

**DOI:** 10.1128/jvi.01529-25

**Published:** 2025-10-02

**Authors:** Khwannarin Khemsom, Ruifan Ren, Junping Han, Camila Perdoncini Carvalho, Eric Matthew Snider, Deyong Zhang, Feng Qu

**Affiliations:** 1 Department of Plant Pathology, The Ohio State University 242626 , Wooster, Ohio, USA; 2 Longping Agricultural College, Hunan University 12569, Changsha, China; 3 Hunan Plant Protection Institute, Hunan Academy of Agricultural Sciences, Changsha, China; 4 Allegheny College 5368, Meadville, Pennsylvania, USA; Iowa State University, Ames, Iowa, USA

**Keywords:** tomato yellow leaf curl virus, geminivirus, rolling circle replication, iteron, Rep, intracellular reproductive bottlenecking, virus evolution

## Abstract

The single-stranded, circular DNA genomes of geminiviruses contain iterated motifs of five to six nucleotides, known as iterons, upstream of the replication protein (Rep) coding region. Iterons were previously found to interact with cognate Rep in a sequence-specific manner, and the iteron-Rep interaction was needed for viral DNA replication. Nonetheless, iterons of closely related viruses often have different sequences, suggesting diversifying selection. To identify selection pressures driving iteron diversification, we constructed tomato yellow leaf curl virus (isolate SH2) mutants in which the iteron motifs were replaced with those of closely related tobacco curly shoot virus (isolate Y35). All mutants replicated in inoculated leaves of *Nicotiana benthamiana*, but some failed to spread systemically. However, the systemic movement defects were mostly rescued by *de novo* mutations. Intriguingly, these *de novo* mutations did not restore the iterons to SH2 sequences. Rather, they likely enabled viral escape from repression exerted by the heterogeneous Y35 iterons in the absence of a matching Rep. These results suggest that iterons probably act as sites of competitive binding by host-encoded transcription factors (TFs) and the cognate Rep. We further speculate that iteron-TF binding commences as soon as viral genomes enter cell nuclei, committing genome copies to Rep mRNA transcription and protein translation, but also blocking them from replication. Conversely, iteron-Rep binding would be possible only after Rep is produced and likely repels TFs from some genome copies, permitting replication initiation. Testing this model through future research should clarify the intricate evolutionary interplays between geminiviruses and their crop hosts and inform novel management strategies.

**IMPORTANCE:**

Geminiviruses are important crop pathogens worldwide for which effective control measures are lacking due to an incomplete understanding of their evolutionary dynamics in infected plants. The current study focuses on a class of short sequence repeats in geminiviral genomic DNA, known as iterons, located immediately upstream of the viral gene encoding replication protein (Rep). Iterons are interesting because, although their positions and repeat patterns are conserved across all geminiviruses, their sequence identities are highly diverse. Our investigations revealed that, contrary to previous reports, the sequence identity of iterons is non-essential for tomato yellow leaf curl virus replication. Rather, they are repressors of replication, and this repression is overcome by their binding with cognate Rep. Future investigations will likely unveil novel targets for more effective management of crop diseases caused by geminiviruses.

## INTRODUCTION

Viruses of the family *Geminiviridae* are among the most common pathogens of crop plants (1), causing devastating losses in staple crops such as cassava, cotton, maize, soybean, tomato, and wheat. These viruses have relatively small, single-stranded (ss), circular DNA genomes, encoding up to a dozen viral proteins (2). Geminiviruses replicate their genomes in host cell nuclei by recruiting a host-encoded DNA-dependent DNA polymerase (DNA Pol). Nevertheless, they do encode an accessory replication protein, designated variously as Rep, C1, AC1, or AL1 depending on viruses under investigation, which recruits DNA Pol to geminiviral DNA. The Rep protein is also responsible for specifying the rolling circle replication mode of these viruses. Specifically, upon entering host cells, the single-stranded, circular DNA genome of a geminivirus, designated as (+) strand, enters the nucleus to prime the synthesis of a complementary (or [−]) strand, thereby creating a double-stranded, circular DNA molecule referred to as replicative form (RF). During rolling-circle replication, the (+) strand of RF is cleaved by Rep, and the (−) strand is used as the template for the synthesis of a linear, single-stranded, multimeric (+) strand, which is subsequently cleaved by Rep into unit genomes. The resulting unit-length linear genomes are subsequently re-circularized by Rep into progeny genomes (2, 3).

**Fig 1:**
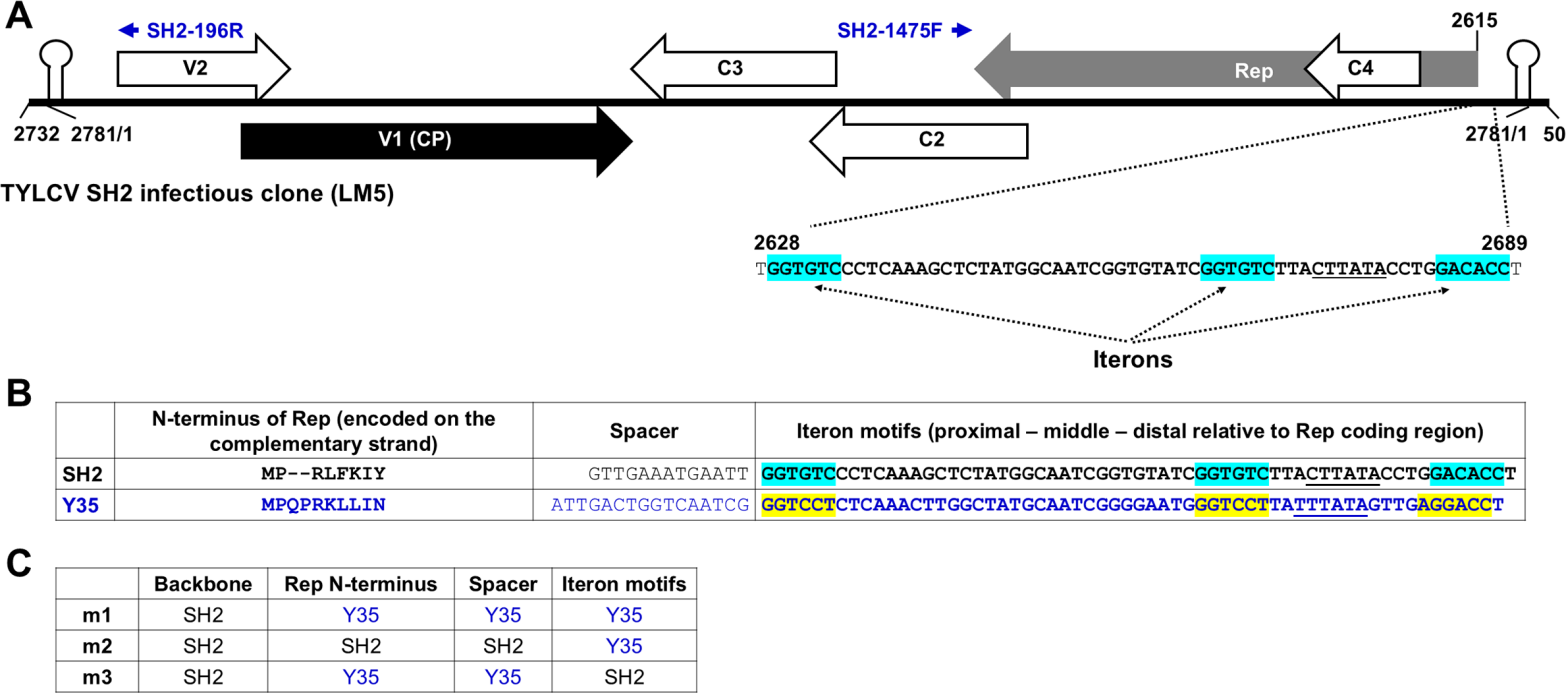
(**A**) Schematic representation of the tomato yellow leaf curl virus (TYLCV) SH2 infectious clone LM5 and the iteron-containing noncoding region. The linearized form of the full-length SH2 genome, plus 50-nt duplications at both ends, was inserted in the binary plasmid pAI101 (7). The large arrows with internal labels of Rep, C4, C2, C3, V1, and V2 denote TYLCV-encoded proteins. The small blue arrows on the top denote a pair of primers (SH2-1475F and SH2-196R) used for specific detection of circularized form of the replication-generated viral DNA. Finally, the 62-nt spanning positions 2628–2689 encompass three SH2 iterons, with the specific iteron motifs painted light blue. (**B**) Comparing the SH2 and Y35 iterons, spacers between iterons and Rep coding sequences, and the first 8/10 amino acid residues of Reps. Y35 nt and aa are in blue fonts, and the Y35 iterons are painted yellow. Note the Reps are coded by the (−) strands of the respective viral genomes. (**C**) Compositions of the three chimeric mutants, m1, m2, and m3.

In addition to encoding proteins, the circular ssDNA genomes of geminiviruses also harbor various *cis*-acting elements. The most important among them is a DNA stem-loop consisting of a double-stranded stem of 11 base pairs (bp) and a single-stranded loop featuring a highly conserved TAATATTAC motif. Inside this motif lies the site of Rep-mediated nicking of circular genomes (TAATATT/AC), a key step required for the initiation of rolling circle replication (4, 5). This DNA stem-loop is indispensable for geminivirus replication and indeed must be duplicated at both ends of a linear, unit-length genome to ensure the infectivity of a geminiviral infectious clone (6) (also see Fig. 1A).

A second class of *cis*-acting motifs, referred to as iterons, are short (five to six nucleotides or nt) sequence repeats located upstream of the Rep protein coding region (8, 9). Iterons typically repeat for three to four times, with at least one of the repeats being antisense to others (9–12). Iterons have been shown to mediate sequence-specific binding with the Rep protein of the same virus (13–16), and this iteron-Rep interaction was found to be needed for successful viral replication. Intriguingly, the iteron-Rep binding required the iteron-containing DNA to be double-stranded (12, 15), suggesting that the double-stranded iteron motifs in RFs are preferred binding sites for Reps (17).

Although iterons are usually conserved among isolates of the same geminivirus species, they are highly divergent among different, though still closely related, virus species. Listed in Fig. S1A are four examples of closely related geminiviruses/isolates, namely tomato yellow leaf curl virus (TYLCV) isolate SH2, tobacco curly shoot virus (TbCSV) isolates Y41 and Y35, and tomato yellow leaf curl Sardina virus (TYLCSV). These viruses share whole genome nt level identities of more than 75% (Fig. S1B, percentages in black fonts) and Rep protein amino acid (aa) level similarities of more than 87% (Fig. S1B, percentages in green fonts). In particular, TbCSV Y41 and Y35 are more than 96% identical at both the whole genome nt level and the Rep aa level. Nevertheless, Y41 and SH2, but not Y41 and Y35, share the same, though differently spaced, iteron sequences (10, 12) (Fig. S1C, the iterons are painted light blue and yellow, respectively). Moreover, iterons of TYLCSV have entirely different sequences (Fig. S1C, iterons are painted purple).

Thus, it appears that iterons, despite their involvement in sequence-specific interactions with cognate Reps, are under rapid diversifying selection. Consistent with this view, Reps, or more specifically the iteron-interacting domains of Reps, also appear to evolve rapidly to maintain the sequence-specific interactions (8, 9, 11). Indeed, comprehensive bioinformatic analyses of large numbers of geminiviruses and nanoviruses identified two separate Rep domains whose aa sequences co-varied with iteron sequences (9, 11) (Fig. S1D). However, it has not been thoroughly examined as to exactly what drives the rapid diversification of iteron motifs and the co-varying iteron-interacting Rep domains.

Here, we report an attempt to identify such selection pressures, using TYLCV SH2 as the model virus and *Nicotiana benthamiana* as the model host (18, 19). TYLCV is a monopartite member of the genus *Begomovirus*, family *Geminiviridae*. Its circular ssDNA genome of approximately 2,800 nt encodes at least six proteins (2, 20, 21). The four proteins (Rep, C2–C4) encoded on the (−) strand of the genome are early expressing, participating in various aspects of viral genome replication, transcriptional activation, and host defense mitigation (Fig. 1A) (2, 22). In particular, Rep is absolutely required for the rolling circle replication of the TYLCV genome (23–25). The two proteins encoded on the (+) strand of the TYLCV genome, known as V1 and V2, are late expressing and function as capsid protein (CP) and suppressor of RNA silencing (26), respectively (Fig. 1A). V2 has also been implicated in viral cell-to-cell movement (27). V1 (CP) is not essential for the replication of TYLCV (28), but is needed for the intra- and intercellular trafficking of the virus (29).

In the current investigation, we modified the TYLCV SH2 genome by replacing its iterons with those of TbCSV Y35 (Fig. 1). We then infected *N. benthamiana* plants with the modified viruses and subjected infected plants to systematic analyses for up to 9 weeks. Identification of *de novo* mutations, and subsequent examinations of these new mutations in plants, led us to conclude that, in the absence of a cognate Rep, iterons act to repress virus replication. Such replicational repression was probably caused by recruitment of host-encoded transcription factors by iterons or other tightly linked sequence motifs. The recruited TFs not only enhanced Rep mRNA transcription to enable rapid accumulation of Rep proteins but also sequestered viral genome copies from replication. While wild-type TYLCV could ease this sequestration through iteron-Rep binding, some of the viral mutants we examined probably escaped the same sequestration through spontaneous mutations that weakened iteron-TF binding. Such mutation-driven escape may prime the diversification of iteron motifs in closely related geminiviruses.

## RESULTS

### Mutants m1 and m3, but not m2, elicit systemic symptoms in *N. benthamiana*

Earlier studies have shown that iterons and Rep of the same geminivirus interact with each other in a highly sequence-specific manner, and this iteron-Rep interaction is necessary for the replication of viral genomes (8, 10–16). However, these studies mostly used viruses with bipartite genomes, or those hosting satellite DNAs, with iteron-disrupting mutations engineered in the non-Rep-encoding genome segments (e.g., DNA-B of bipartite geminiviruses, or satellite DNAs). To determine whether iteron perturbation in *cis* compromises replication of a monopartite geminivirus, we manipulated the genome of TYLCV SH2 to generate three mutants—m1, m2, and m3 (Fig. 1C). In m1, the 97-nt genome section of SH2 encompassing all three iterons, a 13-nt spacer, and the 24 nt encoding the N-terminal 8 aa of SH2 Rep was replaced with the 105-nt counterpart in TbCSV Y35 (Fig. 1B and C). The reason for also exchanging the Rep N-terminus was because this domain, along with another Rep domain approximately 60 aa downstream, was found to co-vary with iteron sequences (9, 11) (Fig. S1D). By contrast, in m2 only the 60-nt iteron region was exchanged, whereas in m3 only the 8-aa (24 nt) Rep N-terminus and 13-nt spacer were replaced (Fig. 1B and C, sections originated from Y35 are highlighted with blue fonts).

**Fig 2:**
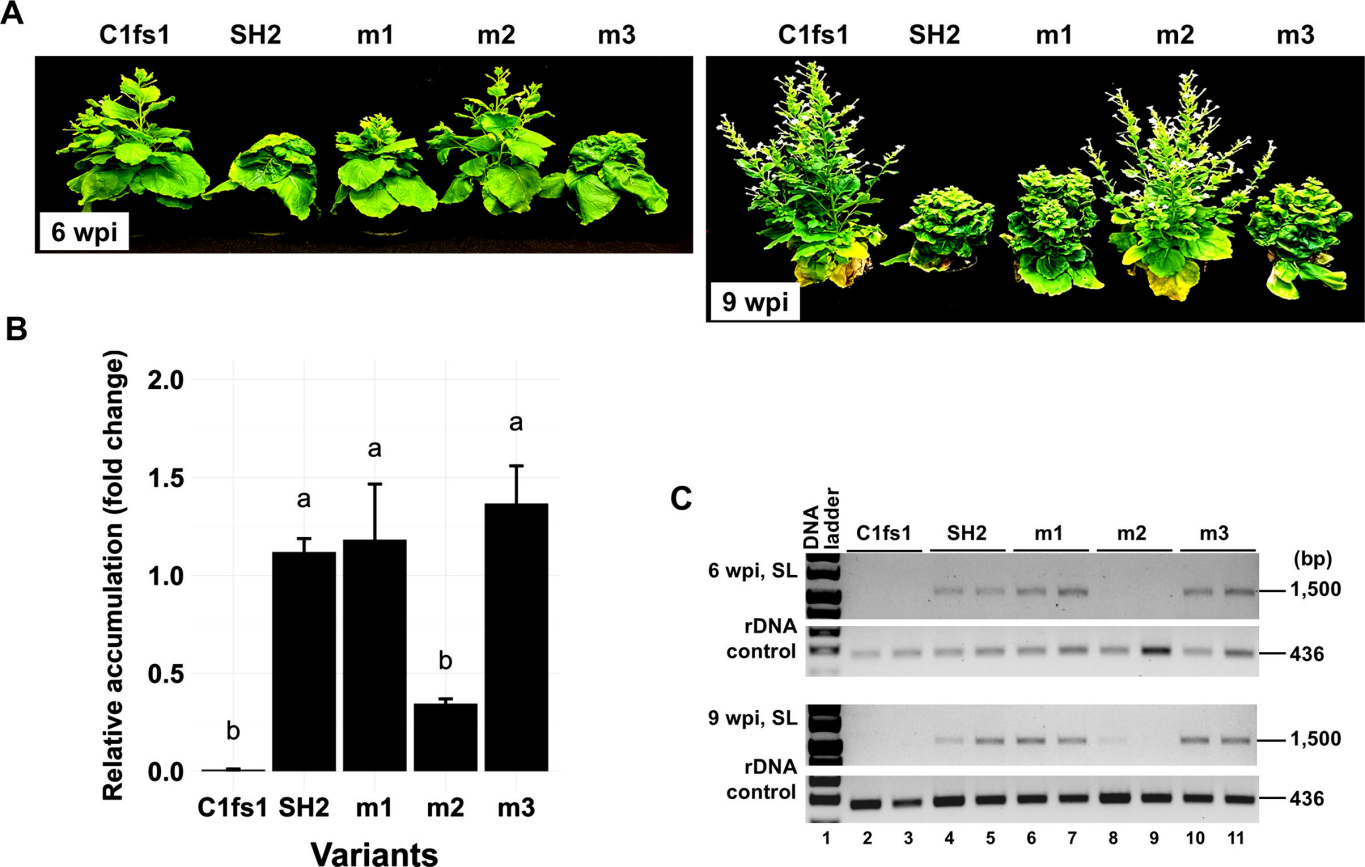
(**A**) Symptoms on *N. benthamiana* plants infected with m1, m2, and m3 mutants. The replication-defective C1fs1 mutant (containing a frameshift mutation in Rep coding sequence) and the wild-type SH2 were used as negative and positive controls, respectively. The two images depicted the plants at 6 and 9 weeks post-inoculation, respectively. (**B**) The relative levels of TYLCV genomic DNA at 1 wpi as assessed with qPCR. Letters above the bars (a and b) signify the statistical significance of the inter-variant differences, with identical letters denoting statistical indifference. (**C**) Presence/absence of viral genomic DNA in the systemically infected leaves at 6 and 9 wpi as assessed with PCR. A 436 bp rDNA fragment was amplified in parallel to serve as a control for DNA quality.

We incorporated three sets of mutations into the wild-type SH2 infectious clone (LM5, Fig. 1A) and tested the resulting mutants in *N. benthamiana*. The m3-infected plants, similar to SH2-infected plants, began to exhibit systemic symptoms at 2 weeks post-inoculation (wpi) (Fig. 2A). The m1-infected plants started to show symptoms by 3 wpi, representing a modest delay of 1 week (Fig. 2A). By contrast, m2 failed to cause visible diseases in any of the six infected plants. The symptom severity differences were consistent with real-time, quantitative PCR (qPCR) detection of viral genomic DNA. As shown in Fig. 2B, m2 genomic DNA levels in 1 wpi inoculated leaves (ILs) were approximately 30% of that of wild-type SH2 control. On the other hand, m1 and m3 genome levels were not statistically different from SH2 (Fig. 2B). Sequence analysis of the PCR products verified that all mutations remained stable in ILs at 1 wpi. When the systemically infected leaves (SLs) were examined at 6 and 9 wpi, both m1 and m3 genomes accumulated to levels indistinguishable from SH2 (Fig. 2C). However, m2 was undetectable at 6 wpi and was present in just one plant by 9 wpi at a very low titer. Thus, among the three mutants, m2 was most debilitated at the systemic infection level.

### Both m1 and m2 mutants incur *de novo* mutations in systemically infected leaves

**Fig 3:**
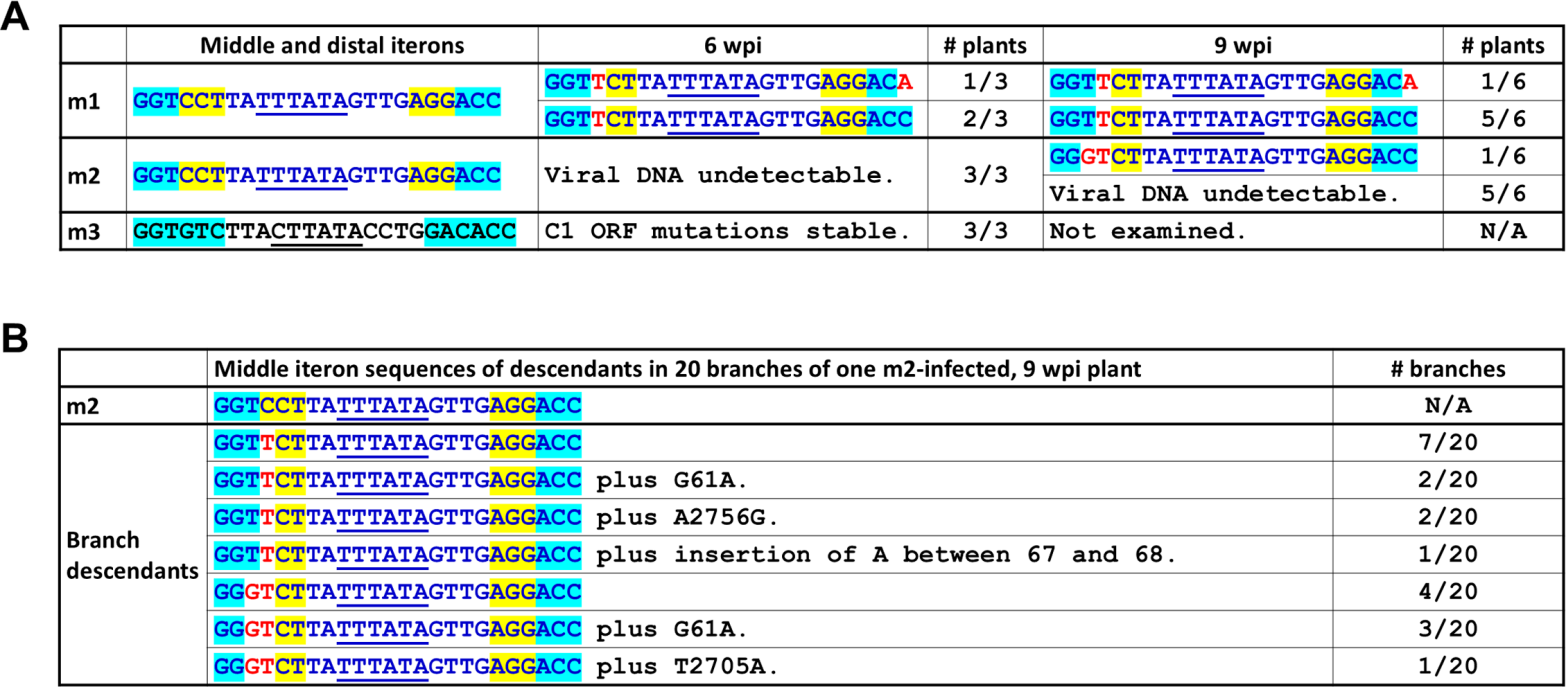
(**A and B**) Sequences of m1, m2, and m3 progeny in systemic leaves at 6 and 9 wpi. Only the sequences spanning the middle and distal iterons are shown. Sequences of Y35 origin are in blue fonts, and those of SH2 origin are in black fonts. Iteron nucleotides of Y35 origin are painted yellow, and those of SH2 origin are painted light blue. The TATA boxes (in antisense orientation) are underlined. Red nucleotides denote *de novo* mutations emerging from infected plants. The extra mutations occurring in different branches all mapped to the intergenic region between Rep and V2 ORFs.

To determine whether the mutations of m1 and m2 were stable in SLs, we next subjected the PCR-amplified genome fragments to sequence analyses. At 6 wpi, viral DNA obtained from three m1-infected plants all contained a new mutation at the fourth position of the middle iteron, changing the Y35-borne GGTCCT motif to GGTTCT (Fig. 3A, *de novo* mutated nt in red font). Furthermore, one of the three DNA samples also contained a second mutation at the sixth position of distal iteron (AGGACC to AGGACA). Remarkably, neither of the *de novo* mutations returned the iteron motifs to the sequences of wild-type SH2 (GGTTCT vs GGTGTC; AGGACA vs GACACC; Fig. 1C). Even more interestingly, at 9 wpi, the first mutation was detected in all six plants analyzed, and the second mutation was also stable in the plant in which it first emerged. Also worth noting was that the original nt (a C) at the fourth position of the middle iteron was undetectable in any of the samples. It should be noted that direct sequencing of the PCR products could mask the presence of original mutations if they are present at very low concentrations. Nonetheless, overall, these results suggest that robust systemic infection does not require the specific sequences of middle and distal iteron motifs to conform to those of either SH2 or Y35.

None of the three m2-inoculated plants examined at 6 wpi yielded detectable levels of viral DNA. However, at 9 wpi, one of the six plants examined contained low levels of viral DNA (Fig. 2C). When subjected to sequence analysis, the PCR-amplified viral DNA contained two mutations at third and fourth positions of the middle iteron, changing GGTCCT to GGGTCT, deviating the motif sequence even further from that of wild-type SH2 (GGTGTC) or Y35 (GGTCCT). To corroborate this result, we then collected leaf samples from 20 different branches of this plant for DNA extraction and PCR detection of viral DNA. As summarized in Fig. 3B, among the 20 branches, 12 contained descendants that harbored the single C-to-T change also found in m1 descendants, whereas the remaining 8 branches contained variants that had the TC-to-GT change. Together, these results showed that when present in the SH2 backbone, the Y35-borne middle iteron incurred *de novo* mutations that rendered it dissimilar from both SH2 and Y35.

Finally, progeny of m3 maintained the original m3 mutations, suggesting that the 10-aa N-terminus originating from Y35 was stable in the SH2 background and had minimal impact on viral systemic infections. Together, these results demonstrated that when the three iteron motifs were exchanged in concert, the middle motif probably hindered SH2 replication, and its adverse impact must be mitigated through *de novo* mutations in order to restore systemic infection to affected viruses. To reiterate, such new mutations did not convert the motif sequence to that of SH2 or Y35, suggesting a relief from repressive activity exerted by Y35 iterons in the absence of the cognate Y35 Rep.

### *De novo* mutations bolster the infectivity of the m1 mutant

**Fig 4:**
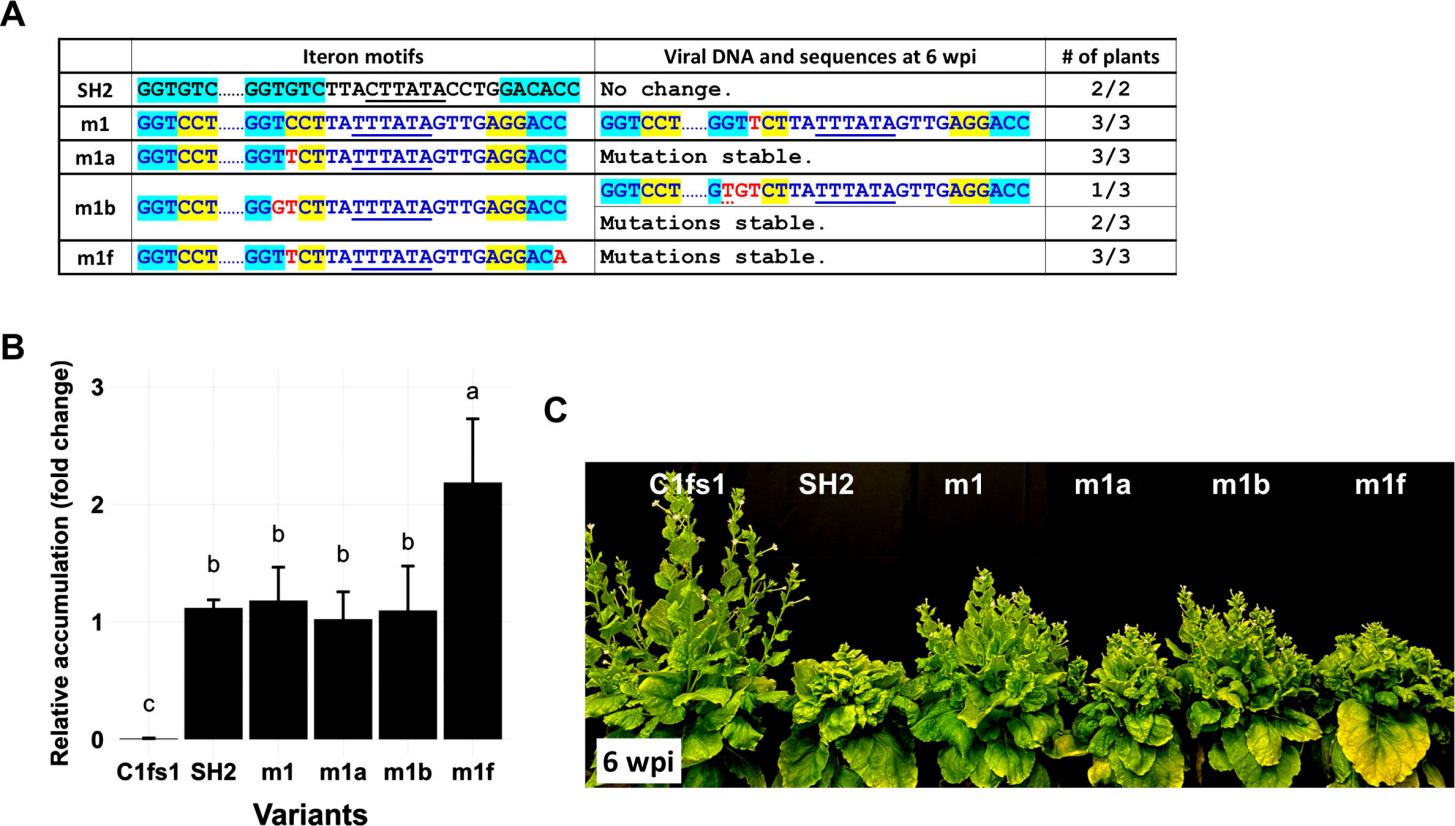
*De novo* mutations identified in m1 and m2 descendants enhance m1 symptoms. (**A**) Iteron portion sequences of new m1-based mutants incorporating the *de novo* mutations and sequences of their descendants at 6 wpi. (**B**) Relative accumulation levels of m1, m1a, m1b, and m1f mutants in IL at 1 wpi as assessed with qPCR. (**C**) Symptoms of infected plants at 6 wpi.

It is worth emphasizing that the original middle iteron of the m1 mutant, of Y35 origin, was not detected in the systemic leaves of plants we examined. We thus speculated that the single C-to-T *de novo* mutation was needed for m1 descendants to spread systemically. To test this, we introduced the C-to-T mutation back into m1 to create m1a (Fig. 4A). For comparison, we also introduced the TC-to-GT mutations recovered from m2 descendants into the m1 backbone, creating m1b (Fig. 4A). Additionally, we incorporated the C-to-A mutation found in the distal iteron into the m1a backbone to create m1f (Fig. 4A). As shown in Fig. 4B, m1a and m1b accumulated genomic DNAs to levels rivaling that of SH2 and m1 in 1 wpi ILs, whereas the m1f accumulation level was significantly higher than even the SH2 wild type. Both m1a and m1f caused symptoms that emerged at about the same time as SH2, thus 1 week earlier than m1 (Fig. 4C). Curiously, the m1b mutant harboring the TC-to-GT double mutations developed symptoms 1 week later than m1a and m1f, causing the infected plants to be taller (Fig. 4C).

When subjected to sequence analyses at 6 wpi, the three new m1-infected plants once again yielded viral progeny that incurred the C-to-T *de novo* mutation (Fig. 4A). Combined with the earlier onset of m1a disease symptoms, the highly reproducible emergence of C-to-T mutation strongly suggested that this mutation was responsible for the systemic infections of m1 descendants. Conversely, the original m1 mutant must have been easily overtaken by the m1a variant containing the C-to-T mutation. Furthermore, this C-to-T mutation, upon emergence in the m1 backbone, was apparently very stable, as it did not evolve further into TC-to-GT. This was unlike the same C-to-T mutation incorporated in the m2 backbone, where it did evolve further into TC-to-GT (see later). Finally, plants infected with the m1f mutant were as severely infected as m1a-infected ones, with both of its mutations remaining stable in plants (Fig. 4B and C). Together, these results indicate that the *de novo* mutations detected in m1 descendants correlated with superior infectivity.

### *De novo* mutations restore infectivity to the m2 mutant without converting the middle iteron to the SH2 motif

**Fig 5:**
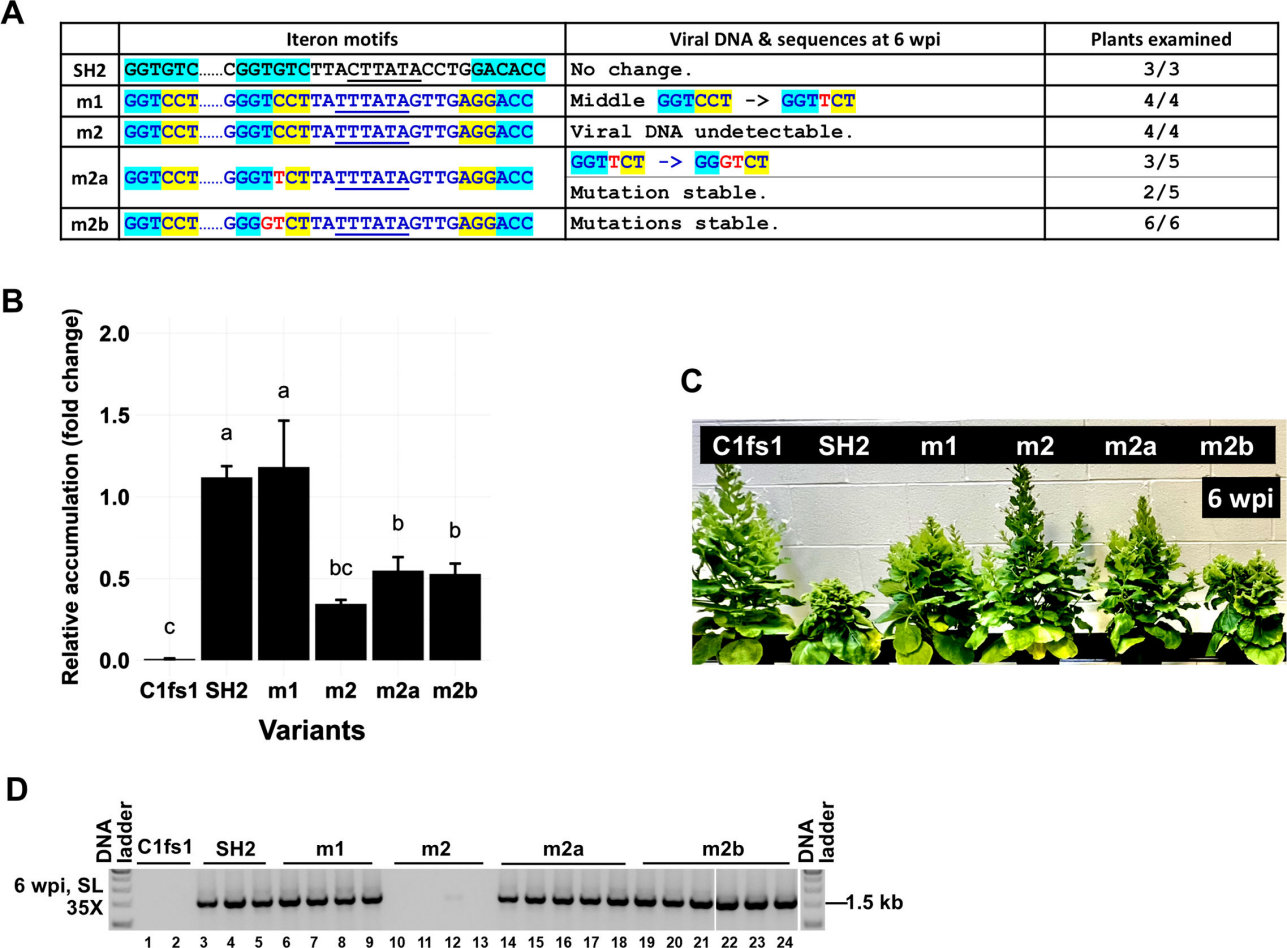
*De novo* mutations identified in m1 and m2 descendants enhance m2 symptoms. (**A**) Iteron portion sequences of new m2-based mutants incorporating the *de novo* mutations and sequences of their descendants at 6 wpi. (**B**) Relative accumulation levels of SH2, m1, m2, m2a, and m2b in IL at 1 wpi as assessed with qPCR. (**C**) Symptoms of infected plants at 6 wpi. (**D**) Presence/absence of viral DNA in SL at 6 wpi as measured with PCR. Note the absence of viral DNA in three of the m2-infected plants and the extremely low accumulation in the fourth.

Unlike the m1-infected plants, those infected with m2 did not have any systemic symptoms, and most of them also failed to accumulate viral genomic DNA (Fig. 2C and 5C). Indeed, even the single plant in which m2 acquired the C-to-T and TC-to-GT mutations did not have visible systemic symptoms. We thus set out to resolve whether these *de novo* mutations also improved the infectivity of the m2 mutant. To this end, the m2a and m2b mutants, carrying the C-to-T and TC-to-GT mutations, respectively, were constructed (Fig. 5A) and used to infect *N. benthamiana* plants. As shown in Fig. 5B, in ILs, both m2a and m2b were able to replicate. However, their accumulation levels were only modestly higher than m2 (not statistically significant). We then examined the systemic leaves through 6 wpi. Unlike m2-infected plants, the m2a- and m2b-infected plants developed clearly visible systemic symptoms (Fig. 5C). Consistently, the TYLCV-specific PCR products obtained from 6 wpi SLs of m2a- and m2b-infected plants reached high levels (Fig. 5D, lanes 14–24).

Since the m1 mutant was also included in the current experiment as one of the controls, we analyzed the sequences of PCR products obtained from m1-infected samples once again. As shown in Fig. 5A, four of four analyzed sequences contained the C-to-T mutation that was identified repeatedly (Fig. 3A, 4A, and 5A). Interestingly, while the C-to-T mutation was very stable in the m1 background (e.g., m1a in Fig. 4), it was less so in the m2 background (m2a). By 6 wpi, the m2a mutant acquired an additional T-to-G change at the adjacent position in three of the five plants, rendering the descendants identical to m2b (Fig. 5A). Consistent with the increased infectivity attributable to the TC-to-GT mutations in m2 (but not m1) descendants, the m2b-infected plants were more stunted than the m2a-infected ones. Furthermore, the m2b mutations remained stable for at least 9 weeks. Collectively, these data indicate that the *de novo* mutations occurring in the m2 genome were necessary and sufficient for the new mutants to gain robust systemic infections.

### The Y35-borne middle motif represses TYLCV systemic infections even without the proximal and distal motifs, and this repression is always relieved by the TC-to-GT mutations

**Fig 6:**
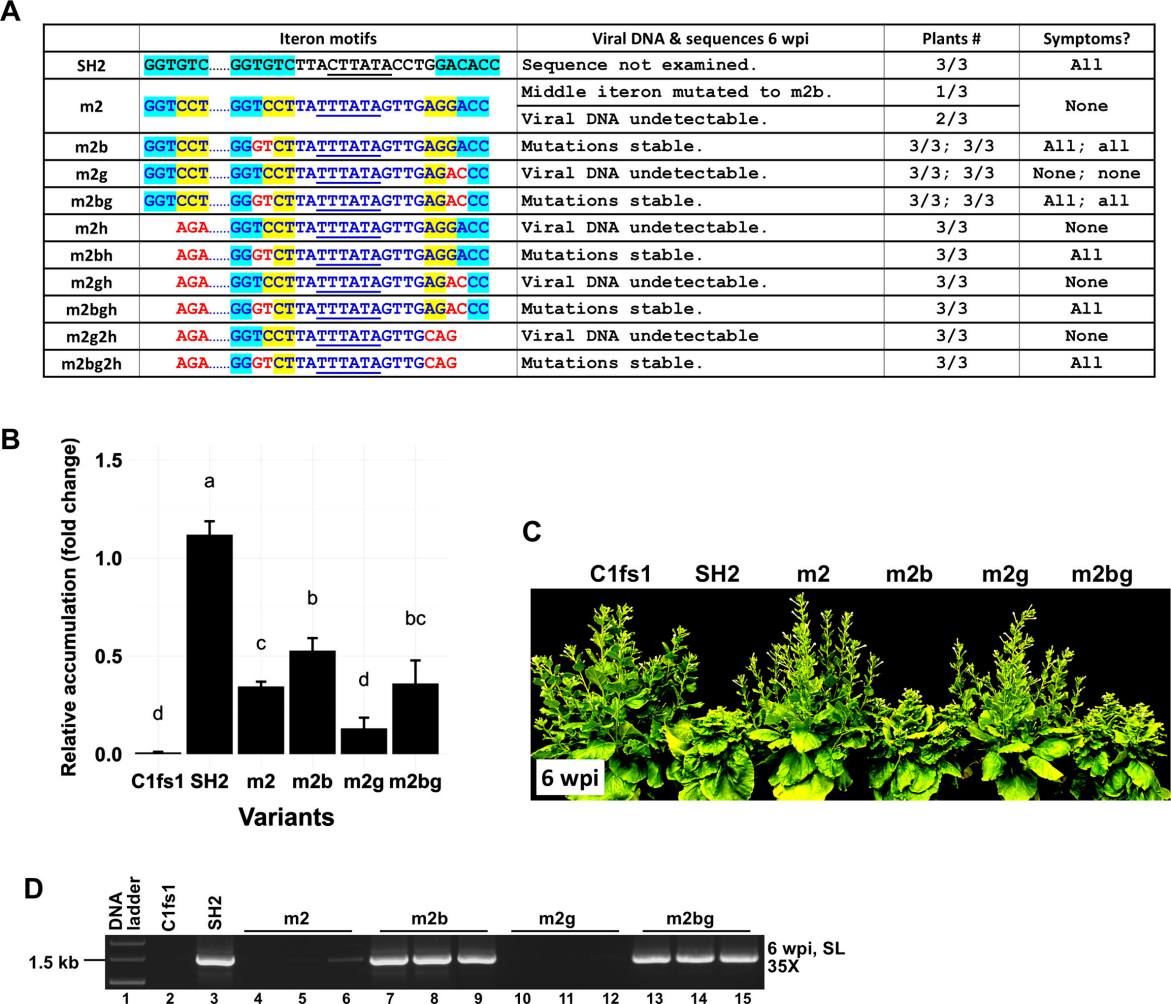
The TC-to-GT mutations of m2b are sufficient to rescue viral replication even if one or both of the flanking iteron motifs (of Y35 origin) are eliminated. (**A**) Iteron portion sequences of the mutants, their absence/presence in 6 wpi SL, stability of the mutations, and SL symptoms. (**B**) Viral DNA levels in 1 wpi IL for a selected set of mutants (m2g and m2b-g) as measured with qPCR. (**C**) Symptoms of representative mutants at 6 wpi. (**D**) Detection of viral DNA in SL with PCR (35 cycles) at 6 wpi.

Given that all three iteron motifs in m2 were of Y35 origin, we next examined if the proximal and distal motifs also contributed to the systemic movement failure of the m2 mutant. To this end, we generated a series of mutants that altered the proximal or distal motif alone or in combination, with or without the TC-to-GT change in the middle motif. As summarized in Fig. 6A, the failure to spread systemically persisted even when one or both flanking motifs were mutated to entirely unrelated sequences (m2g, m2h, m2gh, and m2g2h). While m2g replication in 1 wpi ILs was significantly lower than m2, m2bg containing the TC-to-GT mutations restored replication to m2 levels (Fig. 6B). More strikingly, m2bg, but not m2g, caused robust systemic infections in *N. benthamiana* plants (Fig. 6C). Similar outcomes were also observed with m2bh vs m2h, in which the proximal iteron motif was abolished, as well as m2bgh vs m2gh and m2bg2h vs m2g2h, in which both proximal and distal iteron motifs were mutated (Fig. 6A). Thus, regardless of the sequence identities of proximal and distal motifs, the TC-to-GT mutations in the middle motif alone were enough to restore systemic infections. These results suggest that the flanking motifs were unnecessary for either the middle-iteron-mediated repression or its relief by the TC-to-GT mutations.

### Systemic infection of the m2 mutant is variably bolstered by other mutations at the third and fourth positions of the middle iteron

**Fig 7:**
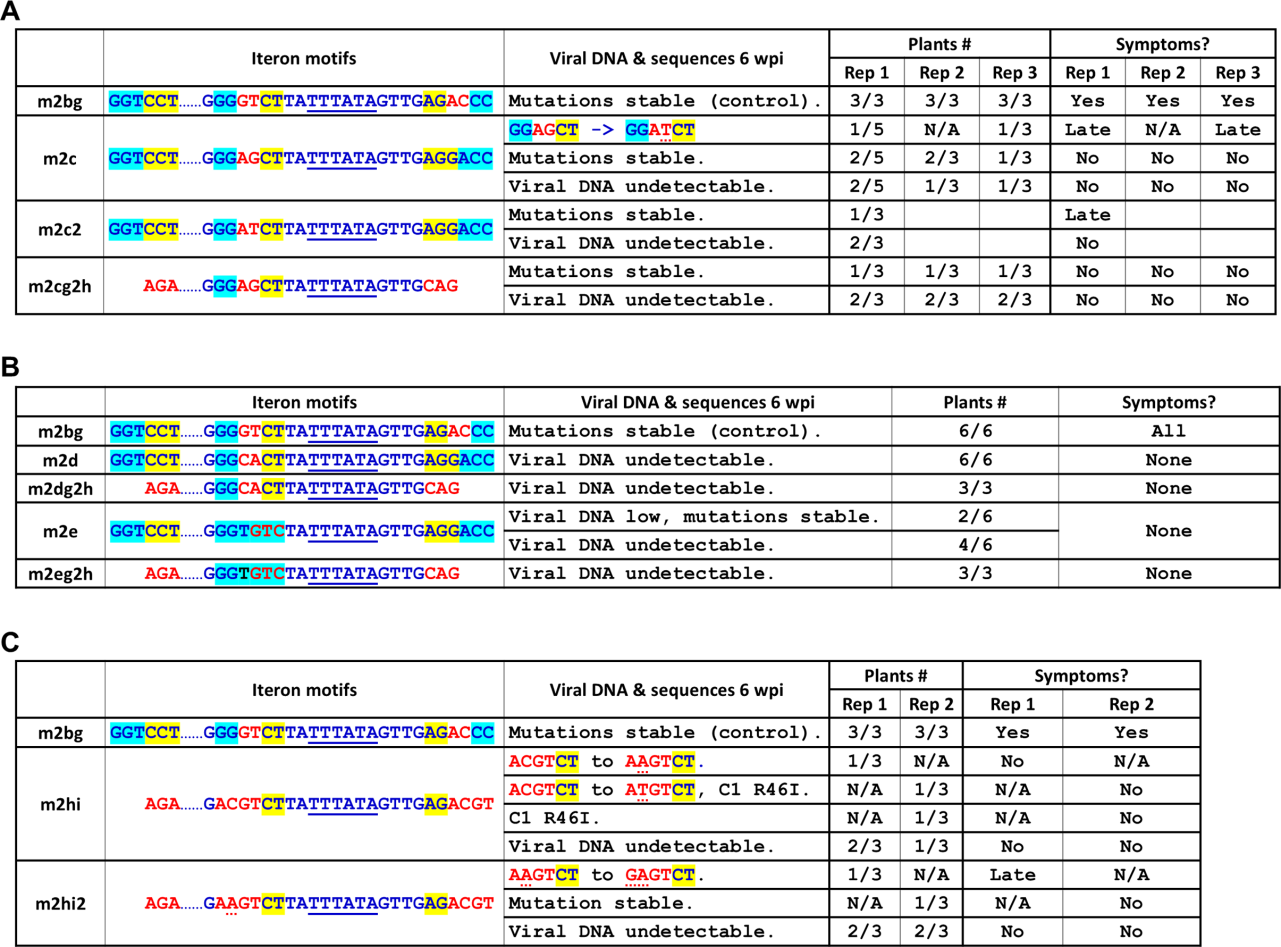
*In planta* fate of more mutants altering the two central nt of the Y35-borne middle iteron, with or without additional manipulations of proximal and distal iterons. (**A**) Infection outcomes of m2c, m2c-g2-h, and m2c2. (**B**) Infection outcomes of m2d, m2d-g2-h, m2e, and m2e-g2-h. (**C**) Infection outcomes of m2h-i and m2h-i2. Images of infected plants were not included because most of the mutants tested here were either symptomless or exhibited mild symptoms.

So far, we showed that the TC-to-GT mutations at the third and fourth positions of the m2 middle iteron caused the resulting m2b mutant to infect *N. benthamiana* plants systemically, leading to clear symptoms indistinguishable from wild-type SH2. The intriguing puzzle was that the exact sequence of m2b middle iteron differed from that of SH2 or Y35 (GGGTCT in m2b as opposed to GGTGTC in SH2, and GGTCCT in Y35). We thus wondered whether substituting the TC doublet within GGTCCT—the Y35-borne m2 middle motif—with two random nts would be enough to restore systemic infection to m2. To resolve this question, we generated three new mutants—m2c, m2d, and m2e. As shown in Fig. 7A and B, m2c and m2d changed the TC doublet to AG and CA, respectively. The m2e mutant altered the last three nucleotides from CCT to GTC so that this motif was now identical to that of SH2 (GGTGTC), though the rest of the iteron-encompassing section was still of Y35 origin. Furthermore, since the proximal and distal iterons were deemed inconsequential in m2b variants, we also tested three additional mutants in which m2c, m2d, and m2e changes were combined with those of m2g2 and m2h, yielding mutants m2cg2h, m2dg2h, and m2eg2h (Fig. 7A and B).

The m2c mutant replicated in local agro-infiltrated leaves (data not shown). When the upper young leaves were assessed at 6 wpi with PCR, viral DNA was detected in three of five, two of three, and two of three plants in three independent repeat experiments (Fig. 7A). Notably, a *de novo* G-to-T mutation at the fourth position was recovered from one plant in the first trial and another in the third trial, changing the middle iteron motif from GGAGCT to GGATCT (Fig. 7A). More interestingly, in both first and third trials, the plants from which the *de novo* mutation was recovered showed delayed symptoms that emerged at 4–5 wpi, whereas all plants containing the original m2c mutants were asymptomatic.

To further assess the impact of this *de novo* mutation, we created and tested the m2c2 mutant harboring this mutation (Fig. 7A). Indeed, the m2c2 mutant caused systemic symptoms in one of three infected plants, though the symptoms were still delayed, only visible after 4–5 wpi (Fig. 7A). Nonetheless, the m2c2 mutations were stable in this plant. Thus, the fourth position of the middle iteron motif converged to a T residue in plants receiving m1, m2, and m2c mutants, even though the respective starting residues were different (C in m1 and m2, G in m2c). By contrast, the third position residue could be a T (m1 progeny), G (m2 progeny), or A (m2c2 progeny). These results indicate that for systemic infections to occur, it was not necessary for the middle iteron to acquire a definitive sequence identity. Finally, the m2cg2h mutant, in which both the proximal and distal iteron motifs (of Y35 origin) were perturbed with mutations, could still be detected in unaltered form in one of three plants in three independent repeats, though none of the mutant-containing plants showed any symptoms (Fig. 7A).

By contrast, the TC-to-CA mutations of the m2d mutant, with or without the flanking Y35 iterons, led to complete failure of systemic infections (Fig. 7B). Curiously, the m2e mutant, in which the SH2 middle iteron motif had been restored, albeit in the middle of Y35 context, accumulated very low levels of viral DNA in just two of the five inoculated plants by 6 wpi (Fig. 7B, the sixth plant died of injury before reaching 6 wpi), with none of the plants showing any symptoms. Furthermore, eliminating the two flanking Y35 iterons led the resulting mutant (m2eg2h, Fig. 7B) to be completely absent from systemic leaves. The m2e and m2eg2h results indicated that restoring the middle iteron alone to the SH2 sequence was insufficient for the virus to regain symptomatic infections. These results contrasted with those of m2b and m2c2, where the departure of the middle motif from the SH2 and Y35 consensuses with merely two nt changes was enough to restore symptomatic infections. Overall, they argue against a critical role of unique sequence identity for iterons. Rather, they support the argument that these *de novo* mutations primarily enabled viral escape from repression conferred by Y35 iterons in the absence of Y35 Rep.

### GG doublet at first and second positions of m2b middle iteron is not essential for viral systemic spread

The TC-to-GT change at third and fourth positions led to an altered motif (GGGTCT) that differed from Y35 (GGTCCT) and SH2 (GGTGTC). Nevertheless, all three motifs still shared the GG doublet at the first and second positions. We next tested whether this GG doublet was needed for systemic infections by mutating them to AC. More specifically, the m2hi mutant changed the proximal, middle, and distal motifs to AGA, ACGTCT, and AGACGT, respectively (Fig. 1C, m2h-i). As a result, none of the three iterons retained the sequences of SH2 (GGTGTC/GACACC) or Y35 (GGTCCT/AGGACC), although the flanking non-iteron sequences were of Y35 origin (Fig. 7C, m2h-i). None of the six plants inoculated in two separate attempts showed any systemic symptoms. However, viral DNA was detected in one plant in the first trial and two in the second trial (Fig. 7C). Interestingly, all three viral DNA samples incurred *de novo* mutations within the middle motif and/or Rep N-terminus (Fig. 7C). In one plant, the ACGTCT was changed to AAGTCT. In another plant, it was changed to ATGTCT, plus an aa change in TYLCV Rep—position 46 arginine (R) was changed to isoleucine (I). Interestingly, the R46I change was also independently detected in another plant (Fig. 7C). Thus, the m2hi mutant appeared to enrich compensatory changes in the Rep protein. Together, these findings suggested that the GG doublet was not always required for viral systemic spread, as none of the *de novo* mutations restored the GG doublet or even just one G.

To test whether some of the *de novo* mutations incurred in m2hi descendants enhanced viral infectivity, we introduced one of the mutations, ACGTCT to AAGTCT, into m2hi to obtain m2hi2. The m2hi2 mutant was still weak, detectable in one of the three plants in each of two independent experiments. Interestingly, another *de novo* mutation was detected in the 6-nt motif in one plant, further changing AAGTCT to GAGTCT, thus restoring one G at the first position. These results suggest a stepwise evolutionary trajectory toward the restoration of at least one of the two Gs.

### Putative iteron-borne, intramolecular DNA secondary structures do not predict the systemic infection differences of the mutants

Results described above demonstrated that while the Y35-borne middle iteron must incur *de novo* mutations to regain systemic infections, the exact nature of the new nt exhibited a certain preference that defies easy interpretation. We hence considered the possibility that the three iteron motifs might fold into intramolecular DNA secondary structures, which could then be differentially perturbed by the mutations introduced (Fig. S2). We thus used the mFold algorithm (http://www.unafold.org/mfold/applications/dna-folding-form.php) to predict potential DNA secondary structures in the iteron-encompassing sequences of SH2 (m3), Y35 (m1, m2), m1a/m2a, m1f, m1b/m2b, m2c, m2d, and m2e. As shown in Fig. S2, the m1 and m2 iterons (of Y35 origin) were indeed predicted to fold into a relatively stable structure (Δ*G* = −5.96 kcal/mol). However, this structure was weakened by m1a, m2a, m2b, m2b, and m2e mutations to similar extents; hence, it could not explain their differences in symptom severities. Furthermore, despite folding into a much weaker structure, the m1f mutant symptoms were indistinguishable from those of m1a. Finally, the m2bg2h mutant, with both flanking motifs deleted, was not expected to fold into stable secondary structures, yet still elicited visible symptoms (Fig. 6A). Thus, the potential secondary structures the iterons could assume in single-stranded genomes do not provide satisfactory explanations for the observed infectivity differences.

## DISCUSSION

### Sequence identity of iteron motifs is not essential for TYLCV replication

The critical importance of iterons with specific nucleotide sequences in geminivirus replication was first reported years ago (13, 15, 16). These earlier studies found that iterons with specific sequence motifs were required for replication of the bipartite tomato golden mosaic virus (TGMV) DNA-B segment, which depended on DNA-A for the replication protein AL1 (13, 15). Similar sequence specificity requirements for monopartite geminiviruses such as TYLCV and TbCSV were inferred from the fact that these viruses likewise harbor reiterated short motifs upstream of the Rep coding region (8, 9, 11). However, whether iteron motifs of monopartite geminiviruses are essential for replication has not been carefully examined. To the best of our knowledge, Xu and colleagues (12) were the first to address this question. They found that mutants of the TbCSV isolate Y35 with any two of the three iterons deleted still infected plants systemically, but a mutant with all three iterons deleted was non-infectious (12).

The current study built on these earlier findings and established that none of the iteron motifs were absolutely required for TYLCV (isolate SH2) replication in initially infected cells. Compared with a mutant of SH2 containing a loss-of-function mutation in Rep (C1fs1), all of our iteron mutants, ranging from m2, in which all three of the SH2 iterons were replaced by their Y35 counterparts, to m2hi, in which all three of them were mutated to even more distinct sequences, were able to replicate to varying levels in agro-inoculated primary leaves. Moreover, some of the mutants, such as m1, m2, m2c, m2cg2h, and m2hi, were detectable in systemic leaves and incurred *de novo* mutations. Yet others, such as m1a/b, m2a/b, m2bg, m2bh, m2bg2h, and m2c2, elicited systemic infections despite extensive perturbation of some or all iteron motifs. Together, our results indicate that specific iteron sequences are not essential for TYLCV replication in infected cells. It will be interesting to find out whether these findings can be reproduced in tomato, the natural host of TYLCV. As kindly reminded by one reviewer of the previous submission, *N. benthamiana* has been found to be highly permissive to many geminiviruses, including TYLCV. Thus, some of our mutants that infected *N. benthamiana* systemically might have a harder time doing so in tomato.

### Heterologous iterons without a matching Rep repress TYLCV replication

An important revelation of our study is that iteron motifs by themselves most likely repress replication of the cognate geminivirus. To explain our reasoning, let us first note that Rep proteins are thought to contain two separate domains mediating iteron binding (11). The first domain corresponds to the N-terminal 8–10 aa, whereas the second maps to aa 66–75 of SH2 Rep or 64–73 of Y35 Rep (Fig. S1). The m1 mutant acquired from Y35, along with the iteron-containing non-coding region, contains the N-terminal iteron-binding domain of Y35 Rep. Thus, a low level of iteron-Rep binding may still occur between Y35 iterons and the matching Rep N-terminus. While the m1 mutant reproducibly elicited systemic symptoms, the descendant viruses always contained the C-to-T *de novo* mutation at the fourth position of middle iteron, converting the motif from GGTCCT to GGTTCT. Put differently, a single mutation within a 105-nt heterologous sequence was enough to rescue robust infections. Therefore, the imported Y35 iterons must have repressed viral replication, necessitating the C-to-T change to escape the repression.

This interpretation is further supported by the fact that the m2 mutant, in which the imported Y35 iterons had to co-exist with the non-matching SH2 Rep, failed systemic infections in most plants. Instead, only the descendants that acquired one or two *de novo* mutations within the middle iteron (GGTCCT to GGTTCT or GGGTCT, m2a and m2b) regained symptomatic infections. Therefore, compared to m1, the further absence of the Y35 Rep N-terminus in m2 caused m2 to be more debilitated, making it necessary to acquire two mutations to relieve the repression imposed by Y35 iterons. We hasten to note that the original m1 and m2 mutants were both able to replicate in cells they first entered, and we surmise that such low-level replication was necessary for *de novo* mutations to emerge and proliferate. Moreover, the possibility of their low-level co-existence in SLs with the dominant, *de novo* mutation-containing derivatives cannot be ruled out. Nevertheless, the original mutants by themselves are unlikely to be competent for efficient systemic spread.

To reiterate, even though the m1 and m2 mutants differed from the wild-type SH2 by sequence stretches of 105 and 60 nt, respectively, acquiring one and two *de novo* mutations within the imported sequences was enough to restore symptomatic infections. Especially, given that both the proximal and distal motifs still retained Y35 sequences in the infection-generated m1 and m2 descendants, it is unlikely that a stimulative role could be regained by altering just one of two nt. It is much more conceivable to foresee such minor changes relieving a certain repressive activity conferred by iterons and/or nearby sequences.

### The *de novo* mutations likely overcome the iteron-mediated repression by evading certain replication-blocking features of iterons and/or nearby DNA sequence(s)

If iterons by themselves repress replication, it can be inferred that in wild-type TYLCV infections, the cognate Rep acted to defeat this repression through iteron-Rep binding, thereby facilitating genome replication (30, 31). How could iteron-mediated repression be defeated when a matching Rep was unavailable? Results with our mutants illustrated that in the absence of a matching Rep, iteron-mediated repression could be overcome by incurring one or two *de novo* mutations within the middle iteron motif. It is worth repeating that the new middle motifs (GGTTCT, GGGTCT, and GGATCT) created through *de novo* mutations did not match that of wild-type SH2 (GGTCTC), thus unlikely to have rescued the original iteron-Rep binding. Further disputing the involvement of a specific sequence was that one of the new middle motifs, GGGTCT, potentiated symptomatic infections even when both proximal and distal iterons (of Y35 origin) were eliminated (the m2bg2h mutant). Conversely, restoring the middle motif to that of SH2 in the Y35 context was insufficient to rescue symptomatic infections (the m2e mutant), even after the Y35 proximal and distal iterons were both eliminated (the m2eg2h mutant). Thus, instead of recreating a new sequence motif to suit SH2 Rep, the *de novo* mutations must have bolstered systemic infections by overcoming repression conferred by the Y35 iterons. As an aside, we consider it unlikely that the mutations we introduced could have disrupted viral movement without compromising viral replication because they were all introduced in the Rep-proximal noncoding region, which is quite distant from the coding sequences of V1 and V2 responsible for intra- and intercellular spread of TYLCV.

How do Y35 iteron motifs repress TYLCV replication? To resolve this puzzle, we considered the possibility that iteron motifs, or other sequence motifs that either overlap with iterons or are tightly linked to them, serve as the binding sites for host-encoded transcription factors. The rationale for this idea is that recruitment of TFs to iterons, which are closely linked to the TATA box of the promoter driving Rep mRNA transcription, likely maximizes Rep production immediately after viral genomes enter host cell nuclei (2). Nevertheless, heavy TF attachment to this iteron-containing genome section likely blocks the same section from being accessed by Rep and other replication-related proteins. Consistent with this idea, iteron motifs are invariably found upstream of the Rep coding sequence (8). Furthermore, their repetition for three to four times within a relatively short stretch closely resembled the arrangement of TF binding sites in the promoters or enhancers of many cellular genes (32–34). For example, the 6-nt auxin-responsive promoter element (AuxRE, TGTCTC) was frequently found multiple times in promoters of auxin-responsive genes, in the form of tandem and/or inverted repeats. Indeed, synthetic promoters with repeated AuxRE motifs were shown to be much more potent than native ones and were frequently used to screen TFs that bind to the motif (32, 35).

Further supporting this idea was the identification of diverse TF binding sites within genomes of various geminiviruses, many of which were shown to mediate transcriptional enhancement of downstream genes (31, 36, 37). Particularly relevant to our discussion is the G-box motif (CACGTG) found in the TGMV DNA-A segment, within the 5′ non-coding region of the TGMV-encoded Rep (AL1). G-box motifs are binding sites for TFs of two large families—the basic helix-loop-helix (bHLH) family and basic leucine zipper (bZIP) family (13, 30, 38). The G-box motif in TGMV DNA-A is separated from iterons by a 28-nt region that contains the TATA box (13). This G-box-plus-TATA-box-plus-iterons region was verified as a functional promoter driving strong transcription of a reporter gene (13, 30, 31). Strikingly, this promoter activity was all but abolished when the G-box motif was mutated, suggesting the involvement of G-box-binding TFs in AL1 mRNA transcription (31). Even more interestingly, this promoter activity was potently repressed by the TGMV AL1 protein through sequence-specific iteron-AL1 binding (30). These observations strongly suggest that TF binding to G-box motif activates AL1 mRNA transcription, whereas Rep binding to iteron motifs represses the same transcription activity, likely by peeling TFs off the promoter through competitive binding (31).

The non-coding region of the SH2 genome encompassing iterons does not contain a G-box motif. However, it does contain two consecutive repeats of a different motif, AATTCAAA, 36 nt upstream of iterons. AATTCAAA is a near-perfect match for the binding sites of three Arabidopsis TFs: TSO1, TCX2, and TCX3 (https://jaspar.elixir.no/search?q=&collection=CORE&tax_group=plants). TSO1, TCX2, and TCX3 are closely related TFs of the CPP (cysteine-rich polycomb-like) family found to play crucial roles in Arabidopsis reproduction by coordinating the cell fate determination in both male and female reproductive organs and controlling stem cell division (39, 40). Separately, the middle iteron motif of SH2, if extended by 2 nt upstream, has the sequence of *TC*GGTGTC, or GACACC*GA* on the complementary strand. Within these 8 nt, TCGGTG/CACCGA is highly enriched in the binding sites of multiple ethylene response factors (ERFs), including ERF011, 019, 037, 038, and 043. Conversely, the Y35 middle iteron, along with two upstream nt, would have the sequence of *TG*GGTCCT/AGGACC*CA*, containing TGGGTCC/GGACCCA, a motif enriched in the binding sites of multiple TCP TFs, including Arabidopsis TCP7, 9, 21, and 22 (https://jaspar.elixir.no/search?q=&collection=CORE&tax_group=plants). TCPs are TFs implicated in diverse processes such as floral symmetry control, branching, lateral organ development, as well as defense responses (41). Notably, the TGGGTCC motif would have been changed by m2b mutations into TGGGGTC, possibly compromising the binding by the same set of TCPs.

### The antagonistic binding of iterons by TFs and Reps hypothesis

**Fig 8:**
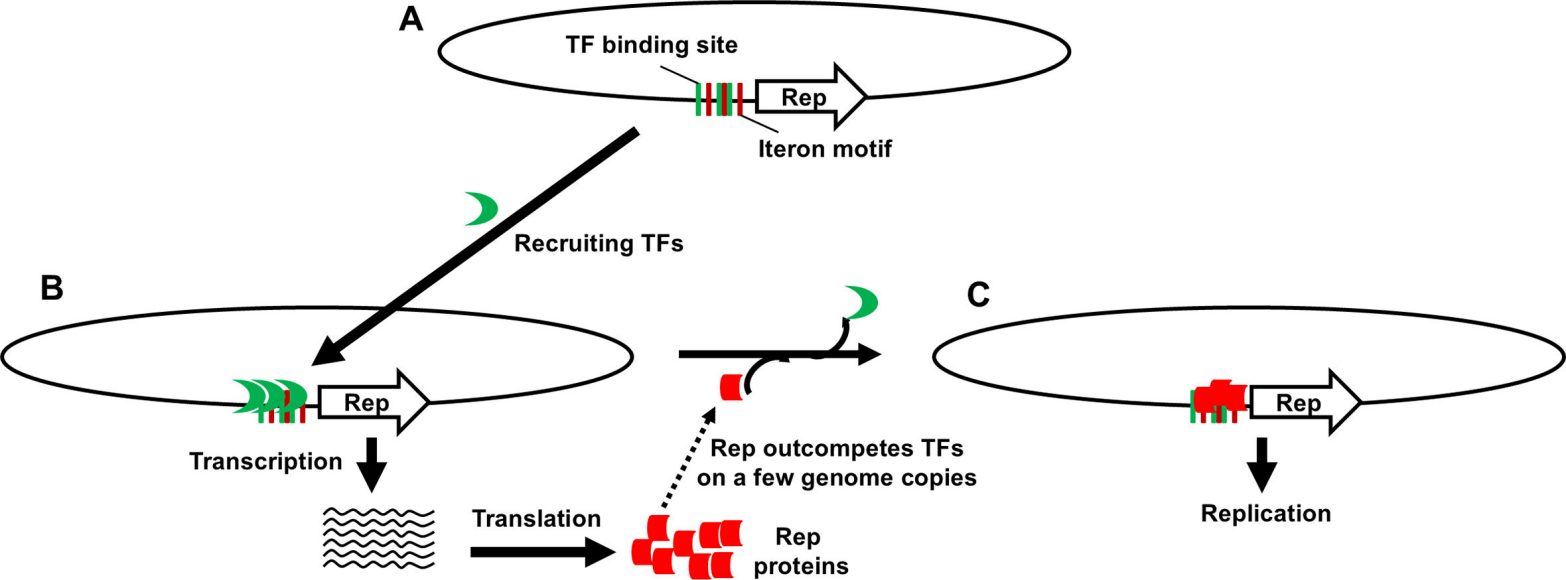
Schematic depiction of antagonistic binding of iterons by TFs and Reps (ABITR). (**A**) Multiple TYLCV genome copies co-entering the same cell nucleus enlist the iteron-proximal TF binding sites to recruit TFs. (**B**) Recruitment of TFs to multiple genome copies activates synchronous Rep mRNA transcription, ensuring rapid accumulation of Rep protein. (**C**) At an appropriate concentration threshold, Rep proteins compete with TFs for iteron motifs and nearby TF binding sites, succeeding in routing a few genome copies per cell to replication.

Together, these observations prompted us to propose the antagonistic binding of iterons by TFs and Reps (ABITR) hypothesis (Fig. 8). ABITR postulates that iterons are tightly linked to, or overlap with, binding sites of certain host-encoded TFs. Geminiviruses evolve TF-binding sites to lure TFs to the Rep promoter, ensuring rapid and efficient production of Rep protein. Yet, occupation of the Rep promoter by TFs also makes the occupied genome copies recalcitrant to replication proteins. This, in turn, selects for iteron motifs nearby, facilitating iteron-Rep interaction that peels off TFs, availing the TF-free genome copies for replication (Fig. 8).

This hypothesis predicts that in the absence of a matching Rep, geminiviral genomes are trapped in a state that is actively transcribed but difficult to transit to replication. Consistent with this prediction, we found that the m2 mutant, harboring Y35 iterons but SH2 Rep, transcribed C1 mRNA to levels exceeding wild-type SH2, yet replicated to just 30% of SH2 (Fig. S3). Similarly, the m1 mutant, though transcribing C1 mRNA to levels three times higher than SH2, replicated to levels barely matching SH2 (Fig. S3). This model further predicts that *de novo* mutations within the TF-binding sites/iterons, such as those in m2b, probably relieved the replicational repression by lessening TF binding to Rep promoters, permitting replication initiation despite the absence of specific iteron-Rep binding.

We hasten to note that the ABITR hypothesis must be rigorously tested with additional investigations to (i) identify the host TFs interacting with the iteron-containing noncoding regions of various geminiviruses; and (ii) demonstrate the competitive binding with iteron-containing DNA by the identified TFs and corresponding Reps *in vitro* and *in vivo*. Finally, it remains to be determined whether this model also applies to bipartite geminiviruses (e.g., TGMV), where Reps must also function in *trans* to replicate the B components of the virus genomes.

### Antagonistic iteron binding by TFs and Reps probably bottlenecks geminivirus replication and drives iteron motif diversification

We previously reported that TYLCV replication was intracellularly bottlenecked, so that among dozens of genome copies that entered a cell, no more than three could initiate replication (19). Such stringent intracellular reproductive bottlenecking is critical for sustaining viral viability as it enables swift purging of defective genome copies harboring lethal and deleterious errors (42–44). However, it is not yet known how geminiviruses like TYLCV establish such intracellular reproductive bottlenecks. The ABITR arrangement, if verified through additional future research, could constitute an early stage of intracellular bottlenecking. This is because, upon cellular entry, the iteron-encompassing TF-binding sites on the Rep promoter should be immediately available for binding with host-encoded TFs. Such promoter-TF binding should be sufficiently stable, hence preventing the subsequently synthesized Rep proteins from de-repressing more than a few genome copies. For the bottlenecks to sustain, there is abundant evidence showing that Rep over-accumulation later during geminivirus cellular infection blocks, rather than facilitates, viral replication. Indeed, Rep overexpression has been widely used to engineer resistance to geminiviruses (45–49). In short, intracellular reproductive bottlenecking of geminiviruses is likely first established with the help of host-encoded TFs and later refortified by over-accumulated Rep proteins.

Conversely, novel TF-binding specificity could emerge if a viral genome copy incurs mutations allowing it to escape TF-imposed reproductive bottlenecking, hence replicating to dominance in cells containing Rep-supplying sister copies. However, such bottleneck-evading cheater copies are bound to accumulate excessive numbers of lethal errors through unconstrained replication. The descendant lineages consisting of such cheaters, upon entering new cells, are expected to either cease replication or acquire additional mutations that together recreate binding sites for different TFs. This then set in motion the evolution of new iteron motifs and new binding specificities in Rep. Such a “Red-Queen” race would explain the rapid diversification of geminivirus iterons.

To summarize, our extensive investigations of TYLCV iterons yielded the surprising revelation that specific iteron sequence motifs are not required for viral replication. Rather, they act as repressors of TYLCV replication, and their interaction with cognate Rep protein serves to overcome this repressive activity. Moreover, in the absence of a cognate Rep, the repressive activity of iterons can also be overcome by incurring relatively few (one or two) *de novo* mutations, thus unveiling a different sequence specificity constraint in addition to iteron-Rep binding. Based on these results, we propose the ABITR model, postulating that sequence motifs in the close vicinity of iterons likely evolve sequence specificity, matching certain host-encoded TFs, thereby recruiting TFs to enhance Rep mRNA transcription and Rep protein production. Once Rep accumulates to a certain concentration threshold, it probably expels TFs from a limited number of viral genome copies through competitive iteron binding, availing these genome copies to bottlenecked replication. Testing predictions of this new ABITR model through future research will likely unveil novel targets for more effective management of crop diseases caused by geminiviruses.

## MATERIALS AND METHODS

### Constructs

The original TYLCV infectious clone (isolate SH2, GenBank accession number: AM282874.1) was kindly provided by Dr. Xueping Zhou of China Institute of Plant Protection (18). The full-length, double-stranded form of the TYLCV genome, with a 50 bp duplication at the 5′ end and another 50 bp duplication at the 3′ end, was subcloned into pAI101, an *Escherichia coli-Agrobacterium tumefaciens* shuttle vector modified from pCambia1300 in our lab (7, 50), resulting in a new TYLCV infectious clone named LM5. The sequence of LM5 encompassing the entire TYLCV DNA (and its modified forms) was verified with Sanger sequencing (51–53).

Most of the mutants were generated by producing the mutation-containing PCR fragments through overlapping PCR (sequences of the primers used are available upon request). The resulting PCR fragments were cloned into the LM5 infectious clone digested with SgsI and Bsp119I. A few mutants were generated with custom-synthesized, mutation-containing DNA fragments (TWIST Biosciences).

### *Agrobacterium* infiltration

All DNA constructs destined for testing in *N. benthamiana* plants were transformed into the electrocompetent *A. tumefaciens* strain C58C1 via electroporation using the AGR setting on the Bio-Rad Micropulser Electroporator. Briefly, 1 µL of the plasmid DNA (100–250 ng) was mixed with 40 µL of agro cells and maintained on ice until electroporation. After electroporation, 900 µL of SOB media was added, and the suspension was incubated at 28°C for 1 hour. Selection was carried out on solid Terrific Broth (TB) media containing rifampicin, gentamycin, and kanamycin. Successful introduction of the plasmid was confirmed using colony PCR. A single colony confirmed to have the desired plasmid was used to inoculate 3 mL of TB liquid media with the same antibiotics and incubated overnight at 28°C. The culture was diluted 1:100 with fresh TB liquid media and incubated under the same conditions for another night. The second culture was centrifuged at 4,000 rpm for 20 min and resuspended in agroinfiltration buffer (10 mM MgCl_2_, 10 mM MES, and 100 µM acetosyringone). All suspensions were diluted to OD_600_ = 0.5 and incubated at room temperature for 3 hours. *Agrobacterium* suspensions were then mixed and introduced into leaves of young *N. benthamiana* plants via a small wound, using a needleless syringe.

### Assessing viral replication in ILs with real-time quantitative PCR

To obtain DNA samples for qPCR analysis, three different plants per treatment group were selected, and from each of them one agro-infiltrated leaf (1 wpi) was chosen to serve as a biological replicate. More specifically, 0.1 g of tissues was cut out of each of the chosen leaves and subjected to DNA extraction using the Quick-DNA Plant Kit manufactured by Zymo Research. The concentrations of the DNA samples were adjusted to 10 ng/µL prior to qPCR. Primers for detecting TYLCV (SH2) genomic DNA were SH2-84R (5′-GAGGATGCAATTTGATTGGTTGACA) and SH2-2693F (5′-GGCTATTTGGTAATTTTGTAAAAGTACATTGC), which together should generate a circular-genome-specific fragment of 173 bp. Control primers for calibrating qPCR conditions were NbITS2-qF (5′-CATTCTCATGGTTGCGGTGC) and NbITS2-qR (5′-CATGACGTTTGTCGCTGTGG), which together amplified an *N. benthamiana* ribosomal DNA fragment of 102 bp. The primers were designed using the NCBI Primer-BLAST tool. The qPCR experiments were carried out using the Luna Universal qPCR Master Mix (New England Biolabs) on a Bio-Rad CFX96 Real-Time System, following the manufacturer’s instructions. Prior to the experiments, the efficiencies of both primer pairs were tested using a protocol recommended by the kit: a five-tube dilution series, each a 10-fold dilution of the prior one, was prepared from a sample known to contain SH2 genomes and subjected to PCR to generate a standard curve through linear regression analysis. The efficiencies were determined to be 87.14% for SH2-84R/2693F and 91.51% for NbITS2-qF/qR. The data obtained were analyzed with R Studio. The threshold cycle (Ct) values of TYLCV variants were normalized against SH2 (wt). Fold change data were calculated with the 2^−ΔΔCt^ method. The fold change data were then subjected to normality test and constant variance test prior to ANOVA and Tukey’s honestly significant difference treatments to determine the statistical significance of the differences among the treatments.

### Detecting *de novo* mutations via Sanger sequencing

The systemic leaf DNA samples were obtained with the Quick-DNA Plant Kit (Zymo Research). For the purpose of Sanger sequencing, a pair of PCR primers that amplified the entire Rep coding region plus the intergenic region between Rep and V2 were used (SH2-1475F and SH2-196R; Fig. 1A; sequences of primers are available upon request). PCR was usually repeated for 35 cycles, and the amplified fragments were sequenced by the OSU Genomics Core Facility.

## Data Availability

All data collected during this study have been described in the text, figures, and supplemental figures. Interested readers are encouraged to contact the authors for further clarification.

## SUPPLEMENTAL MATERIAL

Fig. S1 jvi.01529-25-s0001.TIF Comparison of four closely related monopartite begomoviruses: TYLCV isolate SH2, TbCSV isolates Y41 and Y35, and TYLCSV.

Fig. S2 jvi.01529-25-s0002.TIF Potential DNA secondary structures based on the single-stranded genomes of SH2, Y35, and various mutants, as predicted by mFold.

Fig. S3 jvi.01529-25-s0003.TIF Accumulation levels of C1 mRNA and viral genomic DNA by wild-type SH2 and mutants m1, m2, and m3.

ASM does not own the copyrights to Supplemental Material that may be linked to, or accessed through, an article. The authors have granted ASM a non-exclusive, world-wide license to publish the Supplemental Material files. Please contact the corresponding author directly for reuse.
