# Supplementary figures and images for: Rescue of tomato yellow leaf curl virus mutants harboring heterologous iterons through in planta evolution
Source: J Virol. 2025 Oct 2;99(10):e01529-25. doi: 10.1128/jvi.01529-25 (PMC12548392; doi:10.1128/jvi.01529-25)

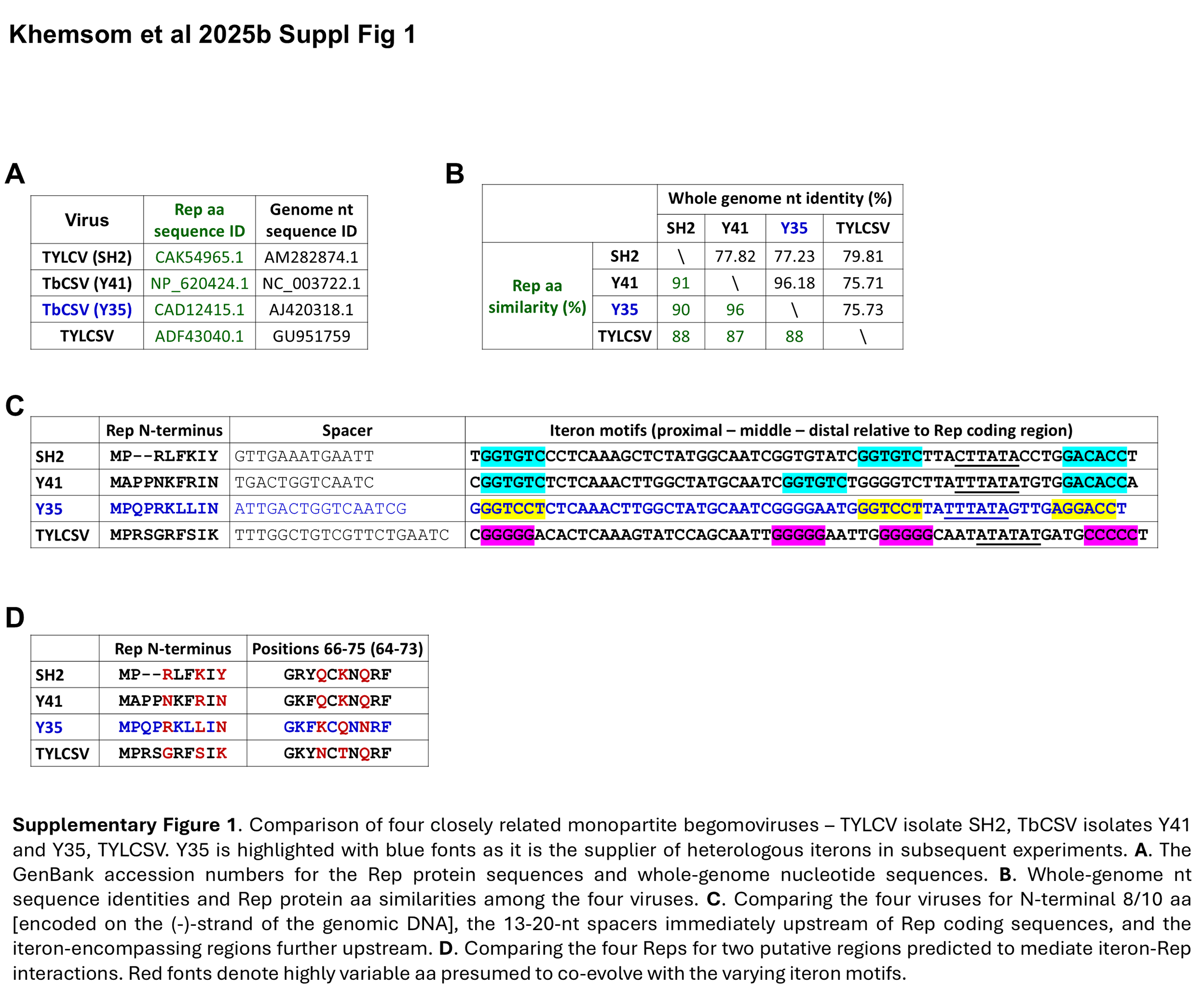

Supplement: Fig. S1 — Comparison of four closely related monopartite begomoviruses: TYLCV isolate SH2, TbCSV isolates Y41 and Y35, and TYLCSV. [file jvi.01529-25-s0001.tif]

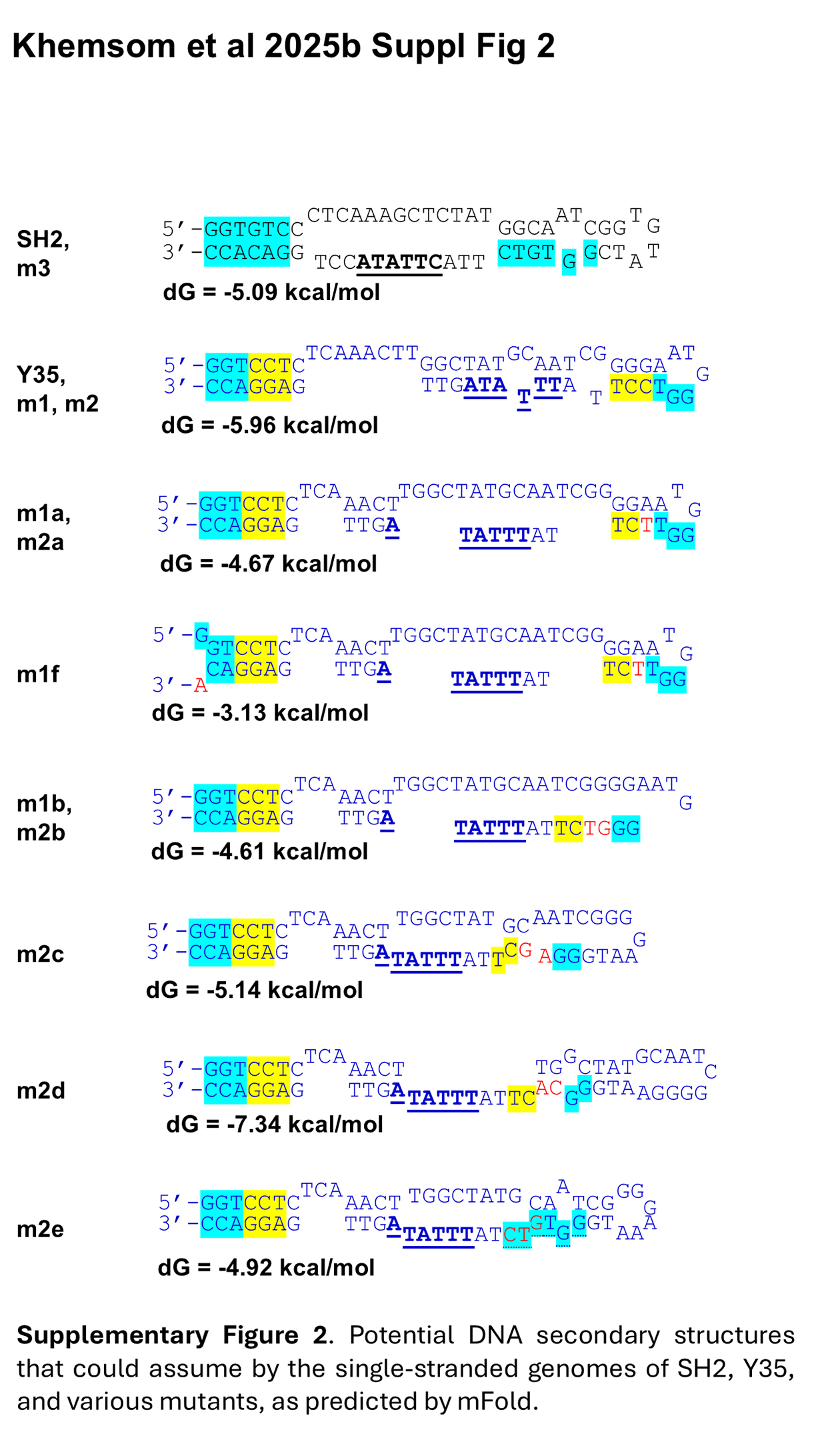

Supplement: Fig. S2 — Potential DNA secondary structures based on the single-stranded genomes of SH2, Y35, and various mutants, as predicted by mFold. [file jvi.01529-25-s0002.tif]

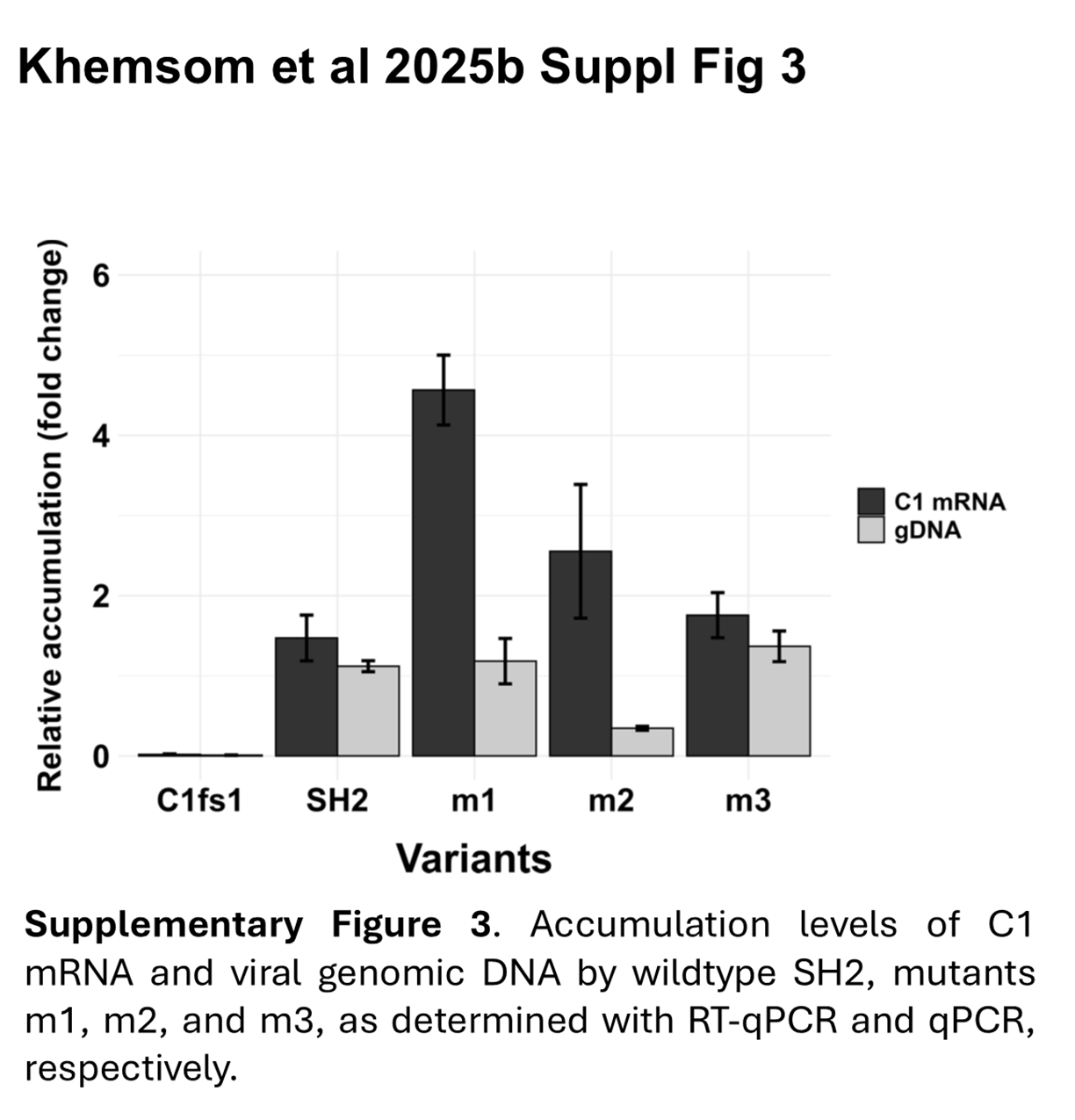

Supplement: Fig. S3 — Accumulation levels of C1 mRNA and viral genomic DNA by wild-type SH2 and mutants m1, m2, and m3. [file jvi.01529-25-s0003.tif]
